# Male genital mutilation (amputation) and its complications: a case report

**DOI:** 10.1186/1756-0500-7-519

**Published:** 2014-08-12

**Authors:** Sam Kaggwa, Moses Galukande

**Affiliations:** Sam Kaggwa Department of Surgery, Makerere University College of Health Sciences, Kampala, Uganda; Moses Galukande, Department of Surgery, Makerere University College of Health Sciences, Kampala, Uganda

**Keywords:** Male genital mutilation, Urethrostomy

## Abstract

**Background:**

Genital losses from ritual attacks are often reported in the media and often discussed in the social media but are hardly reported in medical literature. Male genital mutilation (MGM) refers to permanent modification of the external genitalia that involves ablation of genital tissues.

When found, it is usually as a consequence of poor circumcision skills, auto mutilation/castration or genital injuries caused by attacks or accidents. Male circumcision on its own is widely regarded as a rather safe and acceptable practice which is known to have some health benefits and in keeping with several religious customs as rite of passage. Outside of professional performed circumcision, MGM is usually associated with dark arts and malicious intentions like witchcraft or as a consequence of torture of prisoners of war for information.

**Case presentation:**

In this case we describe a 5-year old Ugandan boy who had his genitals mutilated in bizarre circumstances within a ritual attack. He survived and a urethrostomy was fashioned.

**Conclusion:**

There is need to document more of these cases in order to gather enough information to inform prevention and treatment strategies. Issues of hormonal replacement therapy (HRT) and possible sex change require much debate. These genital sex changing operations should preferably be avoided until a child can fully participate in decision making.

## Background

Genital losses from ritual attacks are often reported in the media and often discussed in the social media but are hardly reported in medical literature [1]. MGM refers to permanent or temporary modification of the external genitalia that involves partial or total ablation of genital tissues or other injury to the male genital organs [2].

When found, it is usually as a consequence of poor circumcision skills, auto mutilation/castration or genital injuries caused by ritual attacks or accidents [3]. Male circumcision on its own is widely regarded as a rather safe and acceptable practice with some health benefits and in keeping with several religious customs as rite of passage. Outside of professionally performed circumcision, MGM is usually associated with dark arts and malicious intentions like witchcraft or as a consequence of torture of prisoners of war for information [4].

In this case we describe a 5-year old Ugandan boy who had his genitals mutilated in bizarre circumstances withina ritual attack. He survived and a urethrostomy was fashioned.

## Case presentation

### History

A five-year-old male was found abandoned by the roadside with hours after his genitalia was crudely severed off, he was bleeding and had herbs strapped onto the perineum.

Given the local community association of the genital mutilation with witchcraft practices, this child was first taken to a traditional healer for wound care. Perhaps with the help of herbs and local pressure application, the acute state of bleeding was controlled. The wound healed with scarring over the meatal stump and scrotal region.

The child was weeks later dropped at the gate of one of the non-governmental organizations (NGO) in the community, upon which the NGO personnel took up care for the child. Reason could have been the traditional healer could not resolve the complications that had occurred concerning difficulty in passing urine. The NGO personnel consulted with local clinics for a period of a year or so, treating the frequent fevers and lower abdominal pain. Upon the realization that the boy’s condition was not resolving, a decision was made to self refer to a higher level facility.

### When the child was presented to hospital

A history of difficulty (excessive strain) in passing urine and urinary frequency were volunteered. Recurrent fevers, abdominal discomfort/pain and a sense of incomplete voiding of urine were reported.

Noteworthy the fevers had been treated as malaria and the child was unable to give information or responded to questions of how the attack happened and who did it and what had transpired thereafter.

He was orphaned (both parents) and taken care by close relatives (whose details were scanty).

### On physical examination

The child was normal looking, healthy but lean with average intelligence and no features suggestive of mental disorders. There was no external genitalia, there was complete healing and scarring with no features of wound infection.

The child was admitted to hospital for reconstruction to ease micturition difficulty. Cosmetic operations and HRT were postponed for a later date given the young age of the patient.

### Urethrostomy procedure

**Figure 1:**
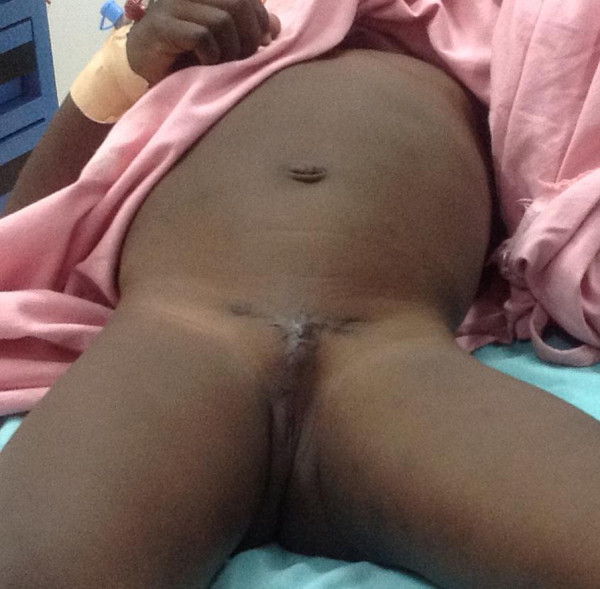
**Shows a T shaped scar with a stenotic orifice.**

**Figure 2:**
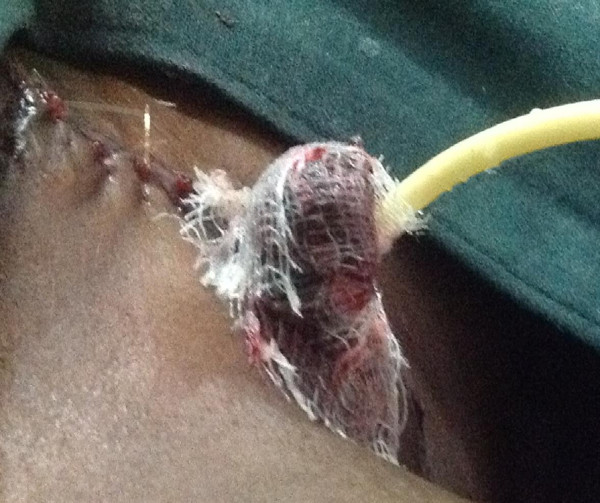
**Shows a reconstructed 3 cm penile stump with a foley's catheter in situ.**

The initial appearance was a T-shaped scar in the perineal area with stenotic urethral orifice in the center (Figure 1). The scar was excised and a 3 cm penile stump though it was devoid of a glans penis (Figure 2).

Half a centimeter of the distal urethra was split open at six o’clock supine position. The side edges were sutured onto the side using 4/0 vicryl, creating a hypospadias. An 8 F Forey’s catheter which was cut to the length of 6 cm was left in the distal urethra as a stent to prevent steriosis as a consequence of tossie swelling or scarring of the neomeatus.

The penile stump was grafted with partial thickness skin graft, harvested from the medial aspect of patient’s right thigh. The wound dressing and urethral catheter left in the situ were removed on the seventh post-operative day.

The graft and urethral orifice healed well. The patient was able to void with a good urinary stream. Six months later, there weren’t any functional (voiding) problems.

## Discussion

We report an extreme case of MGM in bizarre circumstances of a ritual attack. To our knowledge there is no recent (in the past decade or more) scientific publication on the subject in or from Uganda.

MGM, though of rare occurrence and mention, is associated with a more malicious intent and a much worse prognosis compared to female genital mutilation (FGM) as regards psychological trauma, hormonal imbalances and the cosmetic outcome [1–4]. The super incision (dorsal slit) is often refers to a single incision along the upper length of the foreskin from the tip to the corona exposing the glans without removing any tissue. It is the least extreme, and occurs in East Asia [5]. The most wide spread is circumcision, which includes ablation of parts or the entire foreskin. This occurs in all continents as part of a social or religious ritual or medical purposes such as partial HIV prevention in Africa [6, 7].

A more extreme MGM is the sub-incision that exposes the internal urethra ventrally with a longitudinal slit, and was common in aboriginal Australians [8]. The most extreme mutilation is testicular ablation that involves the crushing of or extirpation of the testicles that is documented in South Africa in tribes like Sotho, Khosian. Though varied all genital mutilations share certain features, such as negative sanctions against the mutilated and social benefits for the mutilated are only found in case of a highly public rite of passage.

MGM takes several forms and occurs in about 25% of societies [9]. Only FGM has gained ground, both medical and social groups condemning the practice and advocating for cessation of the practice, that is usually rooted in cultural and spiritual beliefs and strongly entwined to a female’s debut into womanhood and acceptance into the female society once she has undergone the ‘sacred’ ritual. This is common among the Sebei in Uganda and in some countries, FGM is part of a religious ritual like in Somalia, Ethiopia and Eritrea [10, 11].

As a result of the mutilation and scarring in the patient described in this report, there was stenosis of the urethral meatus, resulting in incomplete bladder emptying and consequently resulted in recurrent urinary tract infections. A urethrotomy as was described earlier was performed to relief these difficulties. In the literature several procedures are described for various circumstances [12–15].

Severance of the testicles implies disruption of testosterone supply, and thus posses a question of HRT at puberty so as to commit the patient to external physical features of male gender. In former times gender change was proposed in cases like this, but nowadays this is, often met with much resistance from in the meantime adult victims and their carers. Relevant advocacy organizations strongly recommend that genital operations should be avoided until a child can fully participate in decision making. By contrast a psychological support is offered as early as possible. This matter was not yet discussed in this case with the legal guardian of this boy.

## Conclusion

Such a report contributes to data required to study and understand the epidemiology of ritual induced male genital mutilation with the intent to develop preventive and management strategies.

## Consent

Written informed consent was obtained from the legal guardian of the patient to publish this case report and accompanying images.

## Abbreviations

MGM: Male genital mutilation
FGM: Female genital mutilation
HRT: Hormonal replacement therapy
NGO: Non Governmental Organisation.
